# The Sire Effect on Gestational Length in Wagyu Cattle

**DOI:** 10.3390/vetsci11110551

**Published:** 2024-11-08

**Authors:** Janine de Camargo, Carla Alba, Caroline Gallas, Thales Vogt Kronbauer, Mateus Timbola Mozzato, Dominike Prediger Delazeri, Mariana Groke Marques, Eraldo Lourenso Zanella, Ricardo Zanella

**Affiliations:** 1 ESAN—Veterinary Course, Universidade de Passo Fundo, Passo Fundo 99052-900, RS, Brazil; 110410@upf.br (J.d.C.); 156648@upf.br (C.A.); 51326@upf.br (C.G.); 164017@upf.br (T.V.K.); 140526@upf.br (M.T.M.); 190266@upf.br (D.P.D.); ezanella@upf.br (E.L.Z.); 2 ESAN—Programa de Pós Graduação em Bioexperimentação, Universidade de Passo Fundo, Passo Fundo 99052-900, RS, Brazil; 3 IFC—Instituto Federal Catarinense, Programa de Pós-Graduação em Produção e Sanidade Animal, Concórdia 89703-720, SC, Brazil; mariana.marques@embrapa.br

**Keywords:** wagyu, pregnancy length, genetics, birth weight, beef cattle

## Abstract

This study investigated the factors influencing gestation length in a herd of Wagyu cattle in Rio Grande do Sul, Brazil. Fifty-five multiparous purebred Wagyu cows underwent a Fixed-Time Artificial Insemination (FTAI) protocol using semen from a bull randomly selected from five bulls representing three distinct genetic lines. Following birth, we recorded the calves’ gender, weight, and gestation length. The ANOVA test was employed to assess genetic effects on calf weight and gestation length, with significance set at *p* < 0.05. The 55 inseminations resulted in 52 births, comprising 34 females and 18 males. The average calf weight was 25.9 kg, with 25.2 kg for females and 27.2 kg for males (*p* = 0.38). No significant effect was observed for birth weight concerning the sire’s genetic line (*p* = 0.42), although there was a trend towards an effect from the maternal grandfather (*p* = 0.09). The mean duration of pregnancy was 283.8 ± 5.91 days, with no significant effect of gender on gestation length (*p* = 0.6). However, when evaluating the genetic influences on gestation length, we observed significant effects from the sire’s genetic line (*p* < 0.0001) and the maternal grandfather (*p* < 0.0001). Notably, longer pregnancies resulted in heavier calves (*p* < 0.0001). This is the first study to identify the effects of Wagyu breed lines on pregnancy length.

## Simple Summary

The global Wagyu beef market is expanding, driven by consumer demand for meat with unique characteristics. This breed is known for its exceptional marbling, which enhances flavor and increasingly attracts consumers. Wagyu cattle and their crosses hold significant economic value, justifying investments in genetics to select animals with high-quality meat. Fetal growth plays a crucial role in animal production, as muscle fiber development and adipogenesis occur during this stage. These traits are particularly important for Wagyu cattle. However, little is known about the gestational characteristics of these animals raised in Brazil. This study aims to explore the effects of different Wagyu genetic lines on gestational traits that influence the quality of the offspring. By understanding how genetic lineage impacts the gestational period and the resulting progeny, we can improve mating outcomes in the Wagyu breed. Therefore, it is essential to achieve a balance in assessing the effects of genetic lineage, health, and production traits.

## 1. Introduction

Gestation length (GL), defined as the period from conception to parturition, significantly influences cattle breeding programs and overall production efficiency. Optimal reproductive performance is a key factor associated with economic viability in the beef industry [1]. Research on seven dairy herds in the USA documented an increase in gestation length over time [2]. Environmental factors, along with the age and genetics of dams, can affect gestation length [3]. The age of the cow is a critical determinant of gestation length, with heifers typically experiencing shorter gestation periods compared to older cows [4]. Additionally, a sex effect has been observed, where male calves tend to have longer gestational periods than female calves [3].

Given the implications of gestation length on offspring traits and the recognition that some bulls possess breeding values for gestation length, breeding programs have utilized this information to enhance selection choices and improve postpartum management [5]. Longer gestation periods are associated with a higher frequency of dystocia, while shorter gestation lengths correlate with increased stillbirth rates, particularly in first-parturition scenarios [6]. This underscores the direct relationship between gestation length and calf survivability. Conversely, cows with shorter gestation lengths may experience a higher incidence of postpartum reproductive issues, potentially compromising their longevity and productive performance within the herd [7]. Thus, understanding the effects of gestation length on health and production traits in Wagyu cattle is essential.

Wagyu cattle is a breed that originated in Japan in the last century. Initially, Wagyu were utilized as draft animals for agricultural tasks and were bred for physical endurance [8]. Recently, their remarkable ability to produce high-quality marbled meat has been recognized [9]. Since 1999, Japan has prohibited the export of Wagyu genetic material, resulting in increased levels of inbreeding within the breed [10]. This inbreeding may negatively affect reproductive performance [11,12,13]. Elevated homozygosity often leads to reduced fitness, which can result in weaker calves [14,15]. Several studies have reported the effect of birth weight on the growth efficiency and performance of beef cattle [16,17,18].

Overfeeding pregnant cows during the last trimester of gestation can enhance fat deposition and carcass yield in their offspring, improving adipogenesis without compromising fetal muscle development [19,20]. However, excessive energy intake during pregnancy can adversely affect fetal development, potentially leading to low birth weights [21]. Conversely, maternal malnutrition can negatively impact the fetal loin eye area and calf birth weight [22].

Despite the high economic value of Wagyu cattle and their crosses [23], there is limited information on the reproductive parameters, growth efficiency, and performance of this breed outside Japan. Therefore, the objective of this study was to identify the effects of Wagyu genetic lines on gestation length. This information will enable Wagyu breeders to make accurate predictions regarding expected calving dates for individual animals, thereby informing management decisions.

## 2. Material and Methods

Experimental procedures adopted by this study are in agreement with the Principles of Ethics in Animal Research adopted by the Commission of Bioethics, University of Passo Fundo (UPF, protocol # 007/2021).

### Experimental Design

The work was carried out on a farm in the state of Rio Grande do Sul with Wagyu cattle. Fifty-five multiparous single Pure Breed Wagyu Kuroge cattle, from six sires out of four (*n* = 4) Wagyu Tajima bloodlines, were submitted to a Fixed-Time Artificial Insemination (FTAI) protocol in September of 2022. Cattle were raised in the native field in the summer and in the winter within the rotational grazing system. Cows were fed with an energy mineral supplementation of 150 g/animal/day (MigCorte Energy). The cattle’s average weight was 402 kg, and the body conditions score was 3.1 (1–5) on D0; deworming was performed with levamisole 4.5 mg/kg (SC), Vitamin A 1,000,000 IU/animal single dose (IM).

Estruses and ovulations were synchronized using an intravaginal progesterone device monodose (P4) at 0.5 g, estradiol benzoate (BE) at 2 mg/animal, with D7 0.52 mg/animal of prostaglandin F2-alpha (PGF2α), D9 removal of the P4 device, PGF2α 0.52 mg/animal plus estradiol cypionate (ECP) 2 mg/animal and chorionic gonadotropin equina (eCG) 300 IU/animal.

Estruses were detected using the Estrotect device; after 48 h, it was observed that all females had estrus, and AI was performed using a sanitary AI sheath (IMV Technologies). Each cow was inseminated via AI using semen from 1 out of 5 different bulls from 3 different genetic lines (Itoshigenami (*n* = 2), Itozurodoi (*n* = 1), Itomichi (*n* = 2)). Pregnancy diagnosis was performed by transrectal ultrasonography after 45 days of insemination using the Easi-Scan GO IMV^®^(IMV Technologies, France). Immediately after birth, the zootechnical variables of the animals (gender, weight, and gestation length) were measured. Genetic effects associated with animal weight and gestation length were evaluated using ANOVA in the R statistical program, testing the effect of the sire and maternal grandfather, sex, and animal weight using a multiple regression model.

## 3. Results

**Figure 1:**
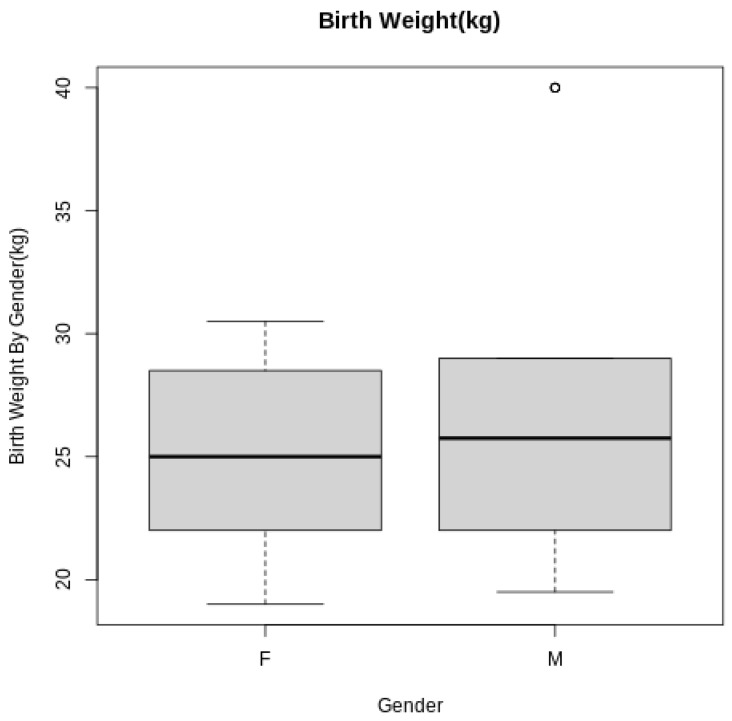
Birth weight associated with gender differences (males = 18, females = 34). The circle in the figure is an animal above the average of the evaluated population.

**Figure 2:**
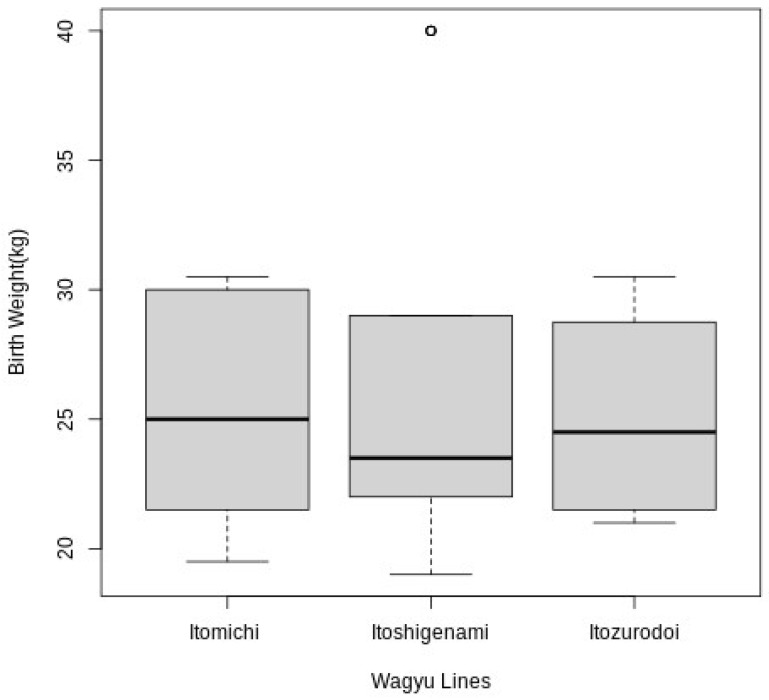
Birth weights associated with different Wagyu lines (Itomichi, *n* = 22; Itoshigenami, *n* = 22; and Itozurodoi, *n* = 8); *p* = 0.34.

The 55 inseminations produced 52 pregnancies (94.45% pregnancy rate), resulting in the birth of 34 females and 18 males. The average weight of the calves was 25.9 kg, and was 25.2 ± 4.03 kg for females and 27.2 ± 7.65 kg for males; no statistical difference was observed among the birth weight for males and females (*p* = 0.38) (Figure 1). Calf birth weights ranged from 21 kg to 28.1 kg across genetic lines, and no statistical difference was observed (*p* = 0.34; Figure 2). Calf birth weights ranged from 20.5 kg to 30.5 kg across (*n* = 6) maternal grandsires (*p* = 0.09).

The average gestational length was 283.8 ± 5.91 days (min = 268 days, max = 295 days). There was no effect of the sex of the calf on the duration of gestation (*p* = 0.6) (males = 284 ± 5.32; females 284 ± 6.26).

**Figure 3:**
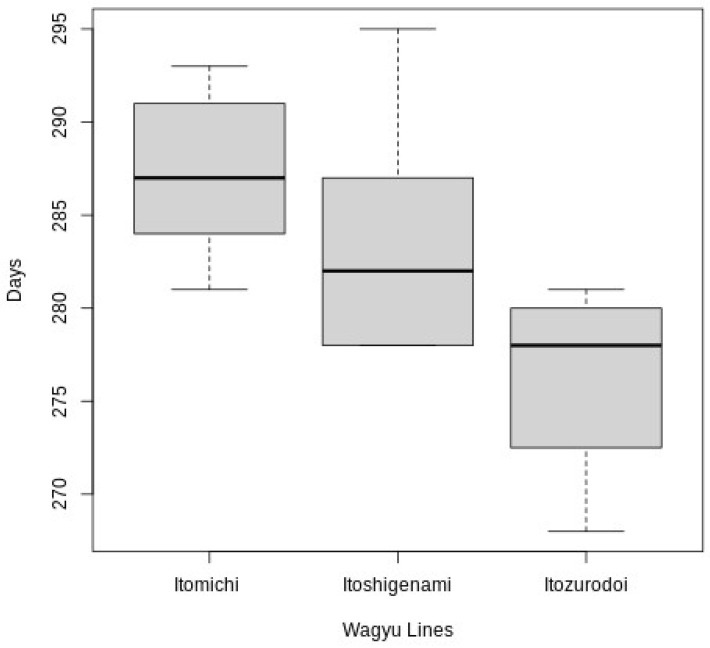
Pregnancy length associated with different Wagyu lines (Itomichi = 22, Itoshigenami = 22, and Itozurodoi = 8).

**Figure 4:**
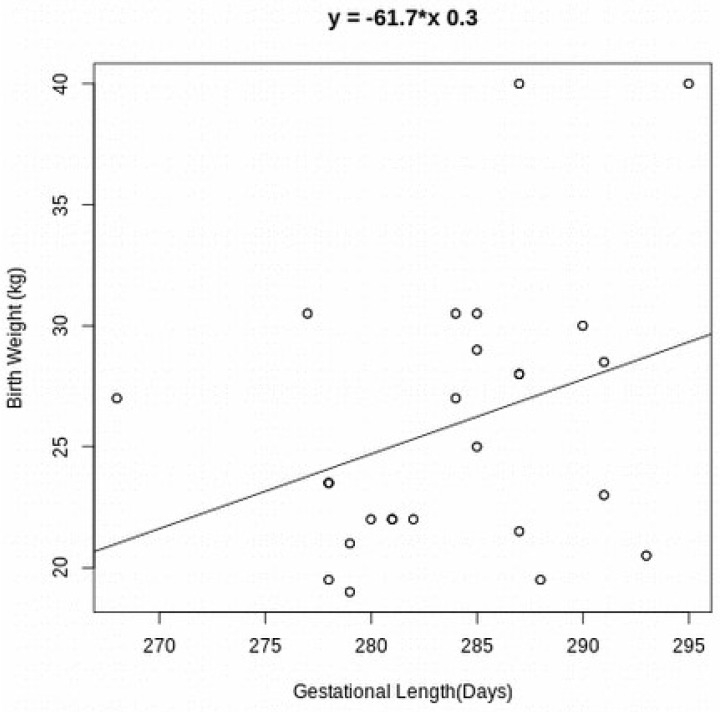
Correlation between gestational length (X-axis) and birth weight (Y-axis) (R^2^ = 0.32, *p* = 0.017). Each circle in the figure represents an animal.

However, when evaluating the genetic effects associated with the length of gestation, testing the sire genetic line and the maternal grandfather line effect on the pregnancy length, we observed a statistical difference, respectively (*p* < 0.0001) (Table 1, Figure 3). In addition to our observation that longer pregnancies tend to produce heavier animals (R^2^ = 0.32, *p* = 0.017), it also indicates the possible effect of gestation time on calf weight (Figure 4).

## 4. Discussion

The global Wagyu beef market is projected to grow from USD 22.64 billion in 2022 to USD 34.87 billion by 2029 at a compound annual growth rate of 6.37% in the period of 2022–2029; the share of Brazil Wagy meat production in this market is very small. However, there is the possibility to produce high-quality meat, or meat at a low cost. Therefore, to produce high-quality meat, attention must be given from the very beginning, especially during fetal development where muscle fiber growth and adipogenesis development take place [24].

Fetal growth, as indicated by birth weight, has an important influence on animal production since lower birth weights are generally associated with increased calf deaths at or near birth. Furthermore, low birth weights also correlate with lower rates of growth and development, affecting meat production and its quality [25]. Calf development and growth are directly associated with birth weight, and therefore, it can be influenced by different factors, including sex, the parity or age of the cow, the breed of the sire, the breed of the dam, heat or cold stress, and nutrition [26]. Understanding which factors can directly interfere with the gestation length is important because it affects the performance of both female and male offspring [5].

Both the sire and dam genetics can contribute to differences in the genetic potential for growth, but it is evident that the maternal effect exerts its influence beyond its contribution to fetal genetic composition [27] because fetal growth appears to have direct and indirect effects mediated by the nutritional status of the dam [21]. In our study, we did not observe the effect of the dam body condition score on the calf’s birth weight. In this regard, nutrient availability to the fetus may be altered by the nutritional status of the mother; however, because all the females were in the same contemporary group or had a similar BCS, this was not a possible observation.

When we tested the effect of the sire genetic line on the birth weight of the calves, we did not observe a difference (*p* = 0.34); this might be an effect of the small population size that was used in our study. Nor were we able to identify the effect of the maternal grandfather (*p* = 0.09). Several articles have identified the effect of the sire’s genetics on the calf’s birth weight because half of the genetic material of the animal is from the sire and the other half from the dam [28].

Even when observing how male calves are 2 kg heavier on average than the females, no statistical difference was observed among their birth weights.

Generally, males tend to have higher birth weights than females [29], but this difference was not significant in our study. With nearly twice as many females born compared to males and the relatively low number of observations made, a lack of significance is not unusual. The coefficient of variation (CV) was 16.0 for females and 28.1 for males, perhaps in part due to differences in the numbers of observations, but females had more uniform birth weights than males.

Interestingly, when we tested the effect of the calf weight on the gestational length, we identified a significant association (*p* = 0.01), indicating that heavier calves have a longer gestation time (R^2^ = 0.32). No effect was identified for gender and gestational length in the wagyu cattle (*p* = 0.6); in addition, no interaction was observed for the gender and birth weight (*p* = 0.18). However, an interaction was observed between the gender and the sire with the gestational length (*p* = 0.02). Finally, when we tested the effect of the sire and maternal grandsire on the gestation length, we were able to identify a strong association between their genetics with the duration of the pregnancy *p* < 0.0001; however, we were not able to identify an interaction between the sire and the maternal grandsire on the pregnancy duration (*p* = 0.485). In dairy cattle and other seasonal cattle breeds, the evaluation of the genetic effect of gestation length has been used as a tool in mating decisions [5,7]. Therefore, understanding factors that can affect the development of the offspring of Wagyu animals is important for decision-making before crossbreeding. Wagyu-sired cattle have an important role in producing superior-quality beef with a greater amount of marbling and a more desirable fatty acid composition because it is assumed to be a trait largely inherited [30,31].

This is the first article to evaluate the effect of the Wagyu genetic line on gestation length and calf birth weight. In addition to this, our study suggests that appropriate nutritional support comprising energy and vitamins is important to manage the success of the reproductive performance in the Wagyu cattle raised in Brazil due to the high metabolism of this particular cattle breed.

## Figures and Tables

**Table 1:** Pregnancy length associated with different Wagyu lines (Itomichi, *n* = 22; Itoshigenami, *n* = 22; and Itozurodoi, *n* = 8); Different letters means a significance difference *p* < 0.05.

Bull Line	Number of Calves	Gestational Length (GL)	Weight (kg)
Itoshigenami	22	283 ± 5.44 ^A^	26.8 ± 7.34
Itozurodoi	8	276 ± 5.74 ^B^	25.1 ± 4.44
Itomichi	22	287 ± 3.74 ^C^	25.3± 4.20

## Data Availability

The datasets analyzed during the current study are not publicly available but are available from the corresponding author on reasonable request.

## References

[1] Diskin M.G., Kenny D.A. (2016). Managing the Reproductive Performance of Beef Cows. Theriogenology.

[2] Silva H.M., Wilcox C.J., Thatcher W.W., Becker R.B., Morse D. (1992). Factors Affecting Days Open, Gestation Length, and Calving Interval in Florida Dairy Cattle. J. Dairy. Sci..

[3] Andersen H., Plum M. (1965). Gestation Length and Birth Weight in Cattle and Buffaloes: A Review. J. Dairy Sci..

[4] Nogalski Z., Piwczyński D. (2012). Association of Length of Pregnancy with Other Reproductive Traits in Dairy Cattle. Asian-Australas. J. Anim. Sci..

[5] Vieira-Neto A., Galvão K.N., Thatcher W.W., Santos J.E.P. (2017). Association among Gestation Length and Health, Production, and Reproduction in Holstein Cows and Implications for Their Offspring. J. Dairy Sci..

[6] Hansen M., Lund M.S., Pedersen J., Christensen L.G. (2004). Gestation Length in Danish Holsteins Has Weak Genetic Associations with Stillbirth, Calving Difficulty, and Calf Size. Livest. Prod. Sci..

[7] Pajohande K., Amirabadi Farahani T., Farsuni N.E. (2023). Increased Incidence of Reproductive Disorders Associated with Short Gestation Length in Holstein Dairy Cows. Theriogenology.

[8] Motoyama M., Sasaki K., Watanabe A. (2016). Wagyu and the Factors Contributing to Its Beef Quality: A Japanese Industry Overview. Meat Sci..

[9] Gotoh T., Joo S.T. (2016). Characteristics and Health Benefit of Highly Marbled Wagyu and Hanwoo Beef. Korean J. Food Sci. Anim. Resour..

[10] Scraggs E., Zanella R., Wojtowicz A., Taylor J.F., Gaskins C.T., Reeves J.J., de Avila J.M., Neibergs H.L. (2014). Estimation of Inbreeding and Effective Population Size of Full-Blood Wagyu Cattle Registered with the American Wagyu Cattle Association. J. Anim. Breed. Genet..

[11] de Marchi F., Lazzaretti R., de Camargo J., Facioli F.L., Zanella E.L., Nacib Jorge-Neto P., Groke Marques M., Caires K.C., Zanella R. (2023). Association between Anti-Müllerian Hormone Levels and Reproductive Parameters in Wagyu Cattle Raised in Brazil. Zygote.

[12] Facioli F.L., Marchi F.D., Marques M.G., Michelon P.R.P., Zanella E.L., Caires K.C., Reeves J.J., Zanella R. (2020). The Outcome and Economic Viability of Embryo Production Using IVF and SOV Techniques in the Wagyu Breed of Cattle. Vet. Sci..

[13] Moura A.R., Santos A.R., Losano J.D.A., Siqueira A.F.P., Hamilton T.R.S., Zanella R., Caires K.C., Simões R. (2023). Evaluation of Sperm and Hormonal Assessments in Wagyu, Nellore, and Angus Bulls. Zygote.

[14] Pekkala N., Knott K.E., Kotiaho J.S., Puurtinen M. (2012). Inbreeding Rate Modifies the Dynamics of Genetic Load in Small Populations. Ecol. Evol..

[15] Pekkala N., Knott K.E., Kotiaho J.S., Nissinen K., Puurtinen M. (2014). The Effect of Inbreeding Rate on Fitness, Inbreeding Depression and Heterosis over a Range of Inbreeding Coefficients. Evol. Appl..

[16] Dowling D.F. (1979). Effect of Birth Weight on Efficiency of Beef Production. Aust. Vet. J..

[17] Greenwood P.L., Cafe L.M. (2007). Prenatal and Pre-Weaning Growth and Nutrition of Cattle: Long-Term Consequences for Beef Production. Animal.

[18] Vaz R.Z., Lobato J.F.P., Restle J., Costa P.T., Eloy L., Costa J.L.B. (2022). Weight at Conception and Gestational Gains in the Efficiency of Beef Cows and Progeny Performance. Acad. Bras. Cienc..

[19] Duarte M.S., Paulino P.V.R., Nascimento C.S., Botelho M.E., Martins T.S., Filho S.C.V., Guimarães S.E.F., Serão N.V.L., Dodson M.V., Du M. (2014). Maternal Overnutrition Enhances MRNA Expression of Adipogenic Markers and Collagen Deposition in Skeletal Muscle of Beef Cattle Fetuses. J. Anim. Sci..

[20] Wilson T.B., Long N.M., Faulkner D.B., Shike D.W. (2016). Influence of Excessive Dietary Protein Intake during Late Gestation on Drylot Beef Cow Performance and Progeny Growth, Carcass Characteristics, and Plasma Glucose and Insulin Concentrations. J. Anim. Sci..

[21] Zago D., Canozzi M.E.A., Barcellos J.O.J. (2019). Pregnant Cow Nutrition and Its Effects on Foetal Weight—A Meta-Analysis. J. Agric. Sci..

[22] Mossa F., Carter F., Walsh S.W., Kenny D.A., Smith G.W., Ireland J.L.H., Hildebrandt T.B., Lonergan P., Ireland J.J., Evans A.C.O. (2013). Maternal Undernutrition in Cows Impairs Ovarian and Cardiovascular Systems in Their Offspring. Biol. Reprod..

[23] Park S.J., Beak S.H., Jung D.J.S., Kim S.Y., Jeong I.H., Piao M.Y., Kang H.J., Fassah D.M., Na S.W., Yoo S.P. (2018). Genetic, Management, and Nutritional Factors Affecting Intramuscular Fat Deposition in Beef Cattle—A Review. Asian-Australas. J. Anim. Sci..

[24] Du M., Wang B., Fu X., Yang Q., Zhu M.J. (2015). Fetal Programming in Meat Production. Meat Sci..

[25] Ferrell C. (1993). Factors Influencing Fetal Growth and Birth Weight in Cattle. Beef Res. Program. Prog. Rep..

[26] Heinrichs A.J., Heinrichs B.S., Harel O., Rogers G.W., Place N.T. (2005). A Prospective Study of Calf Factors Affecting Age, Body Size, and Body Condition Score at First Calving of Holstein Dairy Heifers. J. Dairy. Sci..

[27] Swali A., Wathes D.C. (2006). Influence of the Dam and Sire on Size at Birth and Subsequent Growth, Milk Production and Fertility in Dairy Heifers. Theriogenology.

[28] Coleman L., Back P., Blair H., López-Villalobos N., Hickson R. (2021). Sire Effects on Birth Weight, Gestation Length, and Pre-Weaning Growth of Beef-Cross-Dairy Calves: A Case Study in New Zealand. Dairy..

[29] Rezende E.V., Reis I.J., Campos C.C., Santos R.M. (2020). Influence of Gestation Length, Seasonality, and Calf Sex on Birth Weight and Placental Retention in Crossbred Dairy Cows. Ciênc. Anim. Bras..

[30] Jaborek J.R., Fluharty F.L., Zerby H.N., Relling A.E. (2023). Growth Performance, Carcass Characteristics, and Fatty Acid Composition of Angus- and Wagyu-Sired Finishing Cattle Fed for a Similar Days on Feed or Body Weight Endpoint. J. Anim. Sci..

[31] Lloyd S.S., Valenzuela J.L., Steele E.J., Dawkins R.L. (2017). Genetics of Marbling in Wagyu Revealed by the Melting Temperature of Intramuscular and Subcutaneous Lipids. Int. J. Food Sci..

